# Pseudoaneurysm of the Perforating Peroneal Artery following Ankle Arthroscopy

**DOI:** 10.1155/2018/9821738

**Published:** 2018-11-21

**Authors:** Ichiro Tonogai, Eiki Fujimoto, Koichi Sairyo

**Affiliations:** ^1^Department of Orthopedics Surgery, Institute of Biomedical Science, Tokushima University Graduate School, 3-18-15 Kuramoto, Tokushima 770-8503, Japan; ^2^Department of Cardiovascular Surgery, Institute of Biomedical Science, Tokushima University Graduate School, 3-18-15 Kuramoto, Tokushima 770-8503, Japan

## Abstract

The use of standard anterolateral and anteromedial portals in ankle arthroscopy results in reduced risk of vascular complications. Anatomical variations of the arterial network of the foot and ankle might render the vessels more susceptible to injury during procedures involving the anterior ankle joint. The literature, to our knowledge, reports only one case of a pseudoaneurysm involving the peroneal artery after ankle arthroscopy. Here, we report the unusual case of a 48-year-old man in general good health with the absence of the anterior tibial artery and posterior tibial artery. The patient presented with a pseudoaneurysm of the perforating peroneal artery following ankle arthroscopy for traumatic osteoarthritis associated with nonunion of the medial malleolus. The perforating peroneal artery injury was repaired by performing end-to-end anastomosis. The perforating peroneal artery is at higher risk for iatrogenic injury during ankle arthroscopy in the presence of abnormal arterial variations of the foot and ankle, particularly the absence of the anterior tibial artery and posterior tibial artery. Before ankle arthroscopy, surgeons should therefore carefully observe the course of the perforating peroneal artery on enhanced 3-dimensional computed tomography, especially in patients with a history of trauma to the ankle joint.

## 1. Introduction

The peroneal artery typically originates from the posterior tibial artery (PTA) below the popliteus muscle, running an oblique course toward the fibula. It then divides into the posterior and anterior branches in the inferior segment of the leg [1]. The anterior branch or perforating branch of the peroneal artery pierces the interosseous membrane obliquely and passing anteriorly, runs on the anterior aspect of the distal tibiofibular syndesmosis posterior to the tendon of the peroneus tertius [1]. In the congenital absence of both the PTA and anterior tibial artery (ATA), the peroneal artery with its anterior and posterior branches remains the only source of supply to the dorsal and plantar arterial network [1].

In a pseudoaneurysm of the foot and ankle, the most frequently involved vessel is the ATA [2]. We have previously reported a case of aneurysm of the ATA [3], but a pseudoaneurysm of the peroneal artery, particularly around the ankle joint, is uncommon [4]. Peroneal artery aneurysm around the ankle joint has been reported following ankle sprain [5–10], fracture [11, 12], and ankle arthroscopy [13] and in hemophiliacs [14].

The literature, to our knowledge, reports only one case of a pseudoaneurysm involving the peroneal artery after ankle arthroscopy [13]. Here, we describe an uncommon case of a 48-year-old man with the absence of the ATA and PTA. The patient presented with a perforating peroneal artery pseudoaneurysm following ankle arthroscopy for the posttraumatic osteoarthritis of the ankle accompanied by the nonunion of the medial malleolus. He had undergone debridement of anterior tibial osteophytes with synovectomy using standard anteromedial and anterolateral portals; an end-to-end anastomosis was also performed to repair the injury to the perforating peroneal artery.

## 2. Case Report

This study was approved by the institutional review board of our institution and adhered to The Code of Ethics of the World Medical Association Declaration of Helsinki. The patient provided written, informed consent.

A 48-year-old male farmer in general good health complained of episodes of right ankle pain for 6 years. The pain had worsened approximately 6 months prior to presentation at our hospital. He initially visited a local doctor and was immediately referred to our hospital because of severe pain and plain radiography findings of degenerative changes and osteoarthritis of the right ankle with nonunion of the right medial malleolus.

He acceded to a history of severe right ankle open fracture due to a road traffic accident at age 18 years, although the details were unclear because it was very long time ago. He had undergone an internal fixation procedure at a local hospital, with subsequent healing of the fracture and rescue of his right foot.

**Figure 1:**
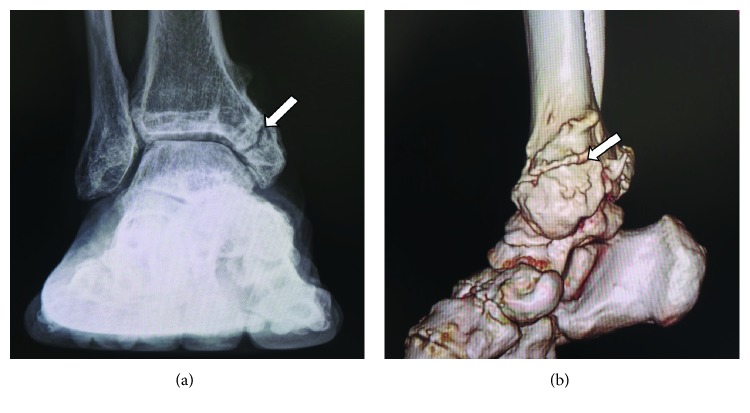
(a) Plain radiography and (b) 3-dimensional computed tomography image indicating nonunion of the medial malleolus. Arrow shows nonunion site of the medial malleolus.

At our hospital, physical examination revealed tenderness at the anterior aspect of the ankle and medial malleolus, with restricted ankle joint range of motion limited to 5° of dorsiflexion. Plain radiography and three-dimensional computed tomography (3D-CT) of the right ankle confirmed that there was no bony bridge formation of the medial malleolar fragment (Figures 1(a) and 1(b)); osteophytes were noted at the anterior edge of the distal end of the tibia with joint space narrowing. We made a diagnosis of posttraumatic osteoarthritis of the right ankle with associated medial malleolar nonunion.

One month later, we performed arthroscopic debridement of the anterior tibial osteophytes with synovectomy and curettage of the medial malleolar nonunion. We recommended ankle fusion but the patient declined, so we performed internal fixation of the medial malleolar fragment using an iliac bone graft. Briefly, noninvasive distraction was applied using an ankle strap and foot traction under general anesthesia and without a tourniquet. An initial skin incision was placed, and the arthroscope was introduced into the articular cavity using a mosquito hemostat. Standard anteromedial and anterolateral portals were created, and an arthroscopic pump was used to induce swelling of the ankle joint. There was extensive damage to the cartilage of the tibial plafond and talar trochlea with exposure of the subchondral bone over the weight-bearing contact area. Osteophytes seen at the anterior edge of the distal aspect of the tibia on subsequent ankle arthroscopy were excised using a bone cutter. Resection of the hypertrophic synovium was done arthroscopically using a 3.0 mm motorized shaver. There was minimal active bleeding from the portals, so the portal sites were closed with nylon sutures. The dorsal pedis artery (DPA) pulse was confirmed palpable following ankle arthroscopy.

A 4 cm longitudinal skin incision was placed along the medial malleolus under a tourniquet. Fibrous tissue was seen between the medial malleolar fragment and the medial distal tibia, and there was visible instability of the fragment. The fibrous tissue was curetted out of the space as much as possible, and the space was filled with the cancellous bone obtained from the iliac bone. Next, the medial malleolar fragment was fixed firmly using two 4.0 mm cannulated cancellous screws, and the skin incision was closed over the medial malleolus using nylon 3-0 sutures.

With the ankle in neutral position, a cast was applied. Sutures were removed 10 days after surgery. We confirmed that DPA pulse was palpable and there was no pulsatile swelling in the anterior lateral aspect of the ankle. The cast was renewed and left in place for a total of 4 weeks. The patient was permitted tolerable weight bearing on the right foot at 3 weeks after surgery. The cast was removed at 4 weeks after surgery and was replaced with an ankle brace.

**Figure 2:**
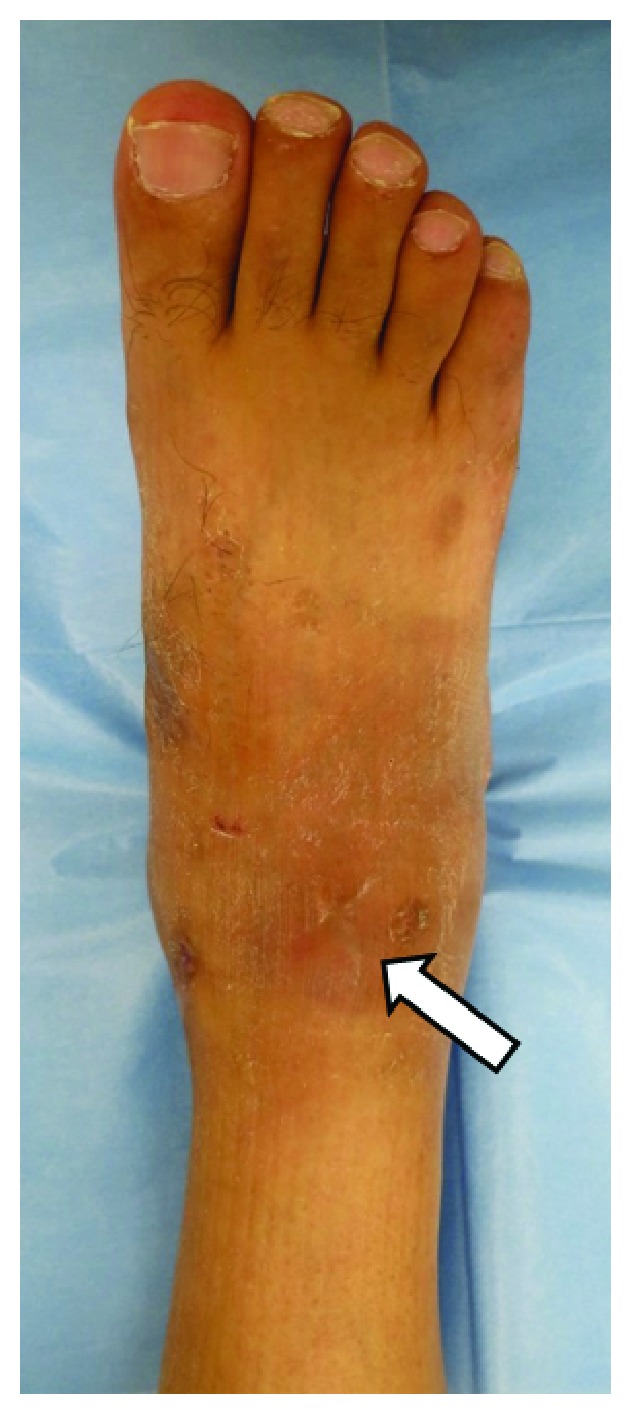
Photograph of the ankle and foot showing swelling around the anterolateral portal (arrow) 4 weeks after ankle arthroscopy.

**Figure 3:**
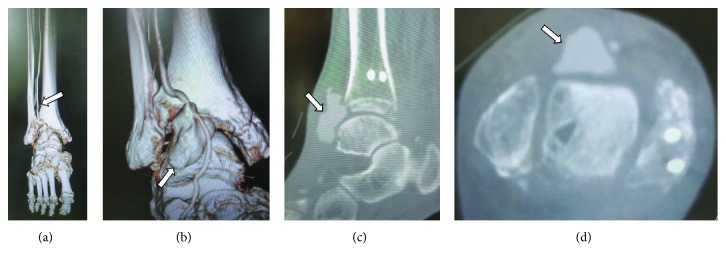
Enhanced three-dimensional computed tomography (CT) images show the perforating peroneal artery (arrow) with the absence of the anterior tibial artery and posterior tibial artery (a) and the pseudoaneurysm of the perforating peroneal artery (arrow) at the lateral aspect of the ankle joint (b). Enhanced CT images also show the size of the pseudoaneurysm (arrow) 15 × 20 × 30 mm on the sagittal (c) and axial planes (d).

**Figure 4:**
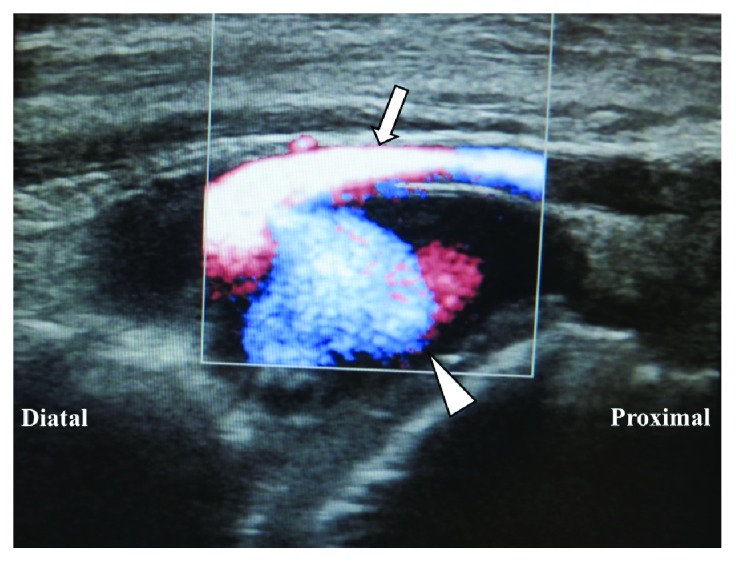
Color and duplex Doppler ultrasonography showing flow through the perforating peroneal artery (arrow) at the anterolateral ankle joint, suggesting a pseudoaneurysm (arrowhead) leaking into the ankle joint. Flow toward and away from the transducer is indicated by red and blue, respectively.

During the early postoperative period and even after weight bearing with cast in situ, the patient had no pain. Following cast removal 4 weeks after surgery, slight tenderness and a pulsatile swelling was noted in the anterolateral aspect of the ankle (Figure 2); the DPA pulse was, however, palpable. The absence of the ATA and PTA was seen on enhanced 3D-CT imaging (Figure 3(a)), with an associated pseudoaneurysm in continuity with the perforating peroneal artery at the level of the anterolateral ankle joint (Figure 3(b)) and with dimensions of 15 × 20 × 30 mm (Figures 3(c) and 3(d)). Color and duplex Doppler ultrasonography displayed a mosaic pattern with pathognomonic “whirling” blood flow and “to-and-fro” motion indicative of a pseudoaneurysm on the wall of the perforating peroneal artery (Figure 4). Our definitive diagnosis was pseudoaneurysm secondary to iatrogenic injury to the perforating peroneal artery at arthroscopy. The patient was subsequently referred to the cardiovascular surgery team at our hospital for further surgical management.

**Figure 5:**
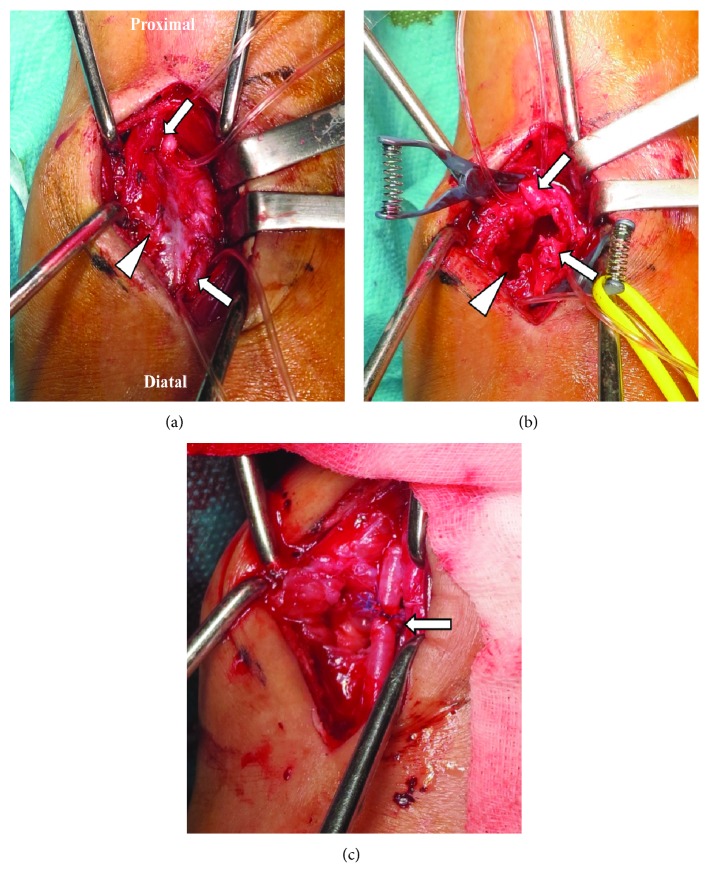
Photographic images taken during repair of the perforating peroneal artery injury. (a) Pseudoaneurysm (arrow) caused by disruption of the perforating peroneal artery wall on the anterolateral side of the ankle joint. Arrow indicates the main trunk of the perforating artery proximally and distally. (b) Pseudoaneurysm is cut (arrowhead) followed by removal of the hematoma-like content within the pseudoaneurysm. Arrow indicates the main trunk of the perforating artery proximally and distally. (c) Repair of the injured perforating peroneal artery wall using end-to-end anastomosis (arrow), without compromising blood supply to the foot.

Five weeks after the ankle arthroscopy, the patient underwent surgery performed by the cardiovascular surgery team. Briefly, a longitudinal incision was placed over the anterolateral aspect of the ankle. There was disruption of the posterolateral 1/3 of the perforating peroneal artery wall with leakage of blood into the ankle, resulting in the pseudoaneurysm (Figure 5(a)). The perforating peroneal artery was isolated and clipped proximally and distally. The pseudoaneurysm was cut, and some hematoma-like content seen within the lumen was removed (Figure 5(b)). The injured arterial walls adjacent each other were sutured using end-to-end anastomosis, and blood flow was preserved distally (Figure 5(c)).

The repair of the perforating peroneal artery injury was successful, and the ankle swelling resolved immediately after surgery. The postoperative course was thereafter uneventful, and the patient returned to his usual farming activities about 4 weeks after the surgical repair of the perforating peroneal artery injury (9 weeks after arthroscopy). Bony union at the medial malleolus was confirmed around 8 weeks after the repair (13 weeks after arthroscopy). The perforating peroneal artery has remained functional, although the patient may require ankle fusion for ankle osteoarthritis in the future.

## 3. Discussion

We report the uncommon case of a 48-year-old man with the absence of the ATA and PTA who underwent arthroscopy for posttraumatic ankle osteoarthritis after which postarthroscopy enhanced 3D-CT imaging revealed a pseudoaneurysm of the perforating peroneal artery. He then underwent repair of the perforating peroneal artery. The English literature, to our knowledge, has only one report of a pseudoaneurysm of the peroneal artery after ankle arthroscopy [13].

The most fatal complication of an untreated pseudoaneurysm of the peroneal artery is the rupture of this vital vessel. This is because the fibrous capsule of a pseudoaneurysm does not have the characteristic three-layered structure of a true aneurysm, and thus it continues to expand until restricted by neighboring structures [3]. This can result in further complications such as hemorrhage into the soft tissue and hemodynamic instability, ankle hemarthrosis, and compartment syndrome in severe cases [15]. We thus opted for surgical treatment, instead of compression or other conservative treatment modalities [16, 17].

Anatomic variations in vascular position may be a factor in the formation of pseudoaneurysms. Regarding ATA deviation, lateral deviation and medial deviation are reported to be found in 5.5% and 3.5% of the population, respectively [18–21]. In the present case, however, this could not be the cause because both the ATA and PTA were absent. In terms of other variations of the arterial network of the foot and ankle, the ATA is absent or filiform in the distal segment and the DPA is supplied by the perforating peroneal artery in about 3% of the population [1]. The absence of ATA and PTA might be congenital in nature in our patient, or the original ATA and PTA might have been injured in the road traffic accident that occurred at age 18 years and the perforating peroneal artery might have developed as collateral circulation. Nevertheless, the peroneal artery was the sole blood supply to the dorsal and plantar arterial network. Consequently, we opted for repair of the peroneal artery and not ligation [22, 23] or embolization [24], in order to preserve blood flow distally. However, we had an alternative plan to reconstruct the perforating peroneal artery using a vein graft if the repair of the perforating peroneal artery with, for example, end-to-end anastomosis was impossible. Surgeons should carefully examine the arterial course and around the ankle and examine for variations of the foot and ankle arterial network on enhanced 3D-CT, especially in patients with previous traumatic ankle injury.

In our case, we could not conclude when exactly the pseudoaneurysm developed because the DPA was palpable shortly after ankle arthroscopy. Furthermore, there were no predisposing factors such as diabetes, malnutrition, immunosuppression, connective tissue disorders, infection, or anticoagulant medication, all of which are associated with a higher risk of pseudoaneurysm because of the compromised vascular wall [25–27]. Also, the perforating peroneal artery was connected to the DPA, so a pinhole injury to the perforating peroneal artery might have occurred when placing or inserting an instrument through the anterolateral portal. Traction of the ankle during osteophyte debridement and synovectomy might be also involved, because structures like vessels can be easily injured due tightness or tautness from traction. In addition, strap placement during joint distraction also might be a factor, because this could compress the artery in the region of the anterior ankle joint capsule [3]. Furthermore, the pseudoaneurysm might have developed and increased in size due to weight bearing after the patient had the right foot in a cast 3 weeks after the arthroscopy. However, distal blood flow was preserved, despite the perforating peroneal artery injury, because the injury was only partial. In case of a complete cut, distal blood flow would not be preserved and necrosis of the foot might have occurred.

A major limitation of this study is the short follow-up period. This patient may need to have surgery-like ankle fusion in the future, although he currently has no ankle pain. A longer follow-up period is important to identify the need for this or other procedures. Also, detailed information about the injury and surgical records were lacking. We tried to retrieve the records from the local hospital but they were no longer available because the injury had occurred a long time ago.

In conclusion, we reported the uncommon case of a 48-year-old man with the absence of the ATA and PTA. He presented with a pseudoaneurysm of the perforating peroneal artery following ankle arthroscopy for posttraumatic ankle osteoarthritis accompanied by nonunion of the medial malleolus and underwent end-to-end anastomosis for the perforating peroneal artery injury. The risk of vascular complications following ankle arthroscopy is much reduced when standard anterolateral and anteromedial portals are used. However, in anatomical variations of vessels, the perforating peroneal artery might be at higher risk of iatrogenic damage during procedures involving the anterior ankle joint. Thus, surgeons should carefully examine for anatomical variations in the course and position of arteries around the ankle on enhanced 3D-CT before ankle arthroscopy, to avoid the development of a pseudoaneurysm, especially in patients with a preceding history of injury to the foot and ankle.

## Abbreviations

3D-CT: 3-Dimensional computed tomography
ATA: Anterior tibial artery
DPA: Dorsal pedis artery
PTA: Posterior tibial artery.

## References

[1] Sarrafian S. K. (1993). *Anatomy of the Foot and Ankle: Descriptive, Topographic, Functional*.

[2] Yu J. L., Ho E., Wines A. P. (2013). Pseudoaneurysms around the foot and ankle: case report and literature review. *Foot and Ankle Surgery*.

[3] Tonogai I., Matsuura T., Iwame T. (2017). Pseudoaneurysm of the anterior tibial artery following ankle arthroscopy in a soccer player. *Case Reports in Orthopedics*.

[4] Sadat U., See T., Cousins C., Hayes P., Gaunt M. (2007). Peroneal artery pseudoaneurysm—a case report and literature review. *BMC Surgery*.

[5] Maguire D. W., Huffer J. M., Ahlstrand R. A., Crummy Jr A. B. (1972). Traumatic aneurysm of perforating peroneal artery. *The Journal of Bone and Joint Surgery. American Volume*.

[6] Marks R. M., Stroud C. C., Walsh D. (1990). Pseudoaneurysm of the lateral malleolar artery after an ankle sprain: case report and review of the literature. *Foot & Ankle International*.

[7] Rians C. B., Bishop A. F., Montgomery C. E., Cahill B. R. (1990). False aneurysm of the perforating peroneal artery: a complication of lateral ankle sprain. A case report. *The Journal of Bone and Joint Surgery. American Volume*.

[8] Sarungi M., Milassin P., Császár J., Sándor L. (1994). Arterial pseudoaneurysm of the ankle after plantar flexion-inversion injury. A rare complication and its non-invasive diagnosis. *Archives of Orthopaedic and Trauma Surgery*.

[9] Bandy W. D., Strong L., Roberts T., Dyer R. (1996). False aneurysm—a complication following an inversion ankle sprain: a case report. *The Journal of Orthopaedic and Sports Physical Therapy*.

[10] Haber L. L., Thompson G., DiDomenico L., Groner T., Glaser J. (2008). Pseudoaneurysm of the perforating peroneal artery after subtalar joint injury: a case report. *Foot & Ankle International*.

[11] Pai V. S. (1999). Traumatic aneurysm of the perforating peroneal artery following ankle fracture. *The Journal of Foot and Ankle Surgery*.

[12] Kurian J., Pillai S. C. B., Chapple D., Frost R. A. (2003). Pseudoaneurysm of peroneal artery following ankle fracture. *Foot and Ankle Surgery*.

[13] Battisti D., Oliva F., Tarantino U., Nicola M. (2014). Pseudoaneurysm of peroneal artery after ankle arthroscopy. *Muscles, Ligaments and Tendons Journal*.

[14] Gopal A., Aranson N., Woo K., Clavijo L., Shavelle D. M. (2013). Recalcitrant peroneal artery pseudoaneurysm in a patient with hemophilia B. *Cardiovascular Revascularization Medicine*.

[15] Brimmo O. A., Parekh S. G. (2010). Pseudoaneurysm as a complication of ankle arthroscopy. *Indian Journal of Orthopaedics*.

[16] van Schaardenburgh P., Steenvoorde P., de Bruïne J. F., Viersma J. H., Warmenhoven P. G. (2003). Thrombotic resolution of a traumatic pseudoaneurysm of the anterior tibial artery after external compression. *The Journal of Trauma*.

[17] Jang E. C., Kwak B. K., Song K. S., Jung H. J., Lee J. S., Yang J. J. (2008). Pseudoaneurysm of the anterior tibial artery after ankle arthroscopy treated with ultrasound-guided compression therapy: a case report. *The Journal of Bone and Joint Surgery. American Volume*.

[18] Huber J. F., Sarrafian S. K. (1993). Anatomy of the foot and ankle. *Anatomy of the Foot*.

[19] Darwish A., Ehsan O., Marynissen H., Al-Khaffaf H. (2004). Pseudoaneurysm of the anterior tibial artery after ankle arthroscopy. *Arthroscopy*.

[20] Mariani P. P., Mancini L., Giorgini T. L. (2001). Pseudoaneurysm as a complication of ankle arthroscopy. *Arthroscopy*.

[21] Yamada T., Gloviczki P., Bower T. C., Naessens J. M., Carmichael S. W. (1993). Variations of the arterial anatomy of the foot. *American Journal of Surgery*.

[22] Ramavath A. L., Cornish J. A., Ganapathi M., Williams D. T. (2009). Missed diagnosis of ankle pseudoaneurysm following ankle arthroscopy: a case report. *Cases Journal*.

[23] Jeffery C. A., Quinn S. J., Quinn J. M. (2014). Pseudoaneurysm of the anterior tibial artery after ankle arthroscopy. *ANZ Journal of Surgery*.

[24] Jacobs E., Groot D., Das M., Hermus J. P. S. (2011). Pseudoaneurysm of the anterior tibial artery after ankle arthroscopy. *The Journal of Foot and Ankle Surgery*.

[25] Kotwal R. S., Acharya A., O’Doherty D. (2007). Anterior tibial artery pseudoaneurysm in a patient with hemophilia: a complication of ankle arthroscopy. *The Journal of Foot and Ankle Surgery*.

[26] Dhillon M. S., Bali K., Prabhakar S. (2012). Differences among mechanoreceptors in healthy and injured anterior cruciate ligaments and their clinical importance. *Muscles, Ligaments and Tendons Journal*.

[27] Williams J. C., Roberts J. W., Yoo B. J. (2010). Dorsalis pedis artery pseudoaneurysm after Lisfranc surgery. *Journal of Orthopaedic Trauma*.

